# Late infantile form of multiple sulfatase deficiency with a novel missense variant in the *SUMF1* gene: case report and review

**DOI:** 10.1186/s12887-023-03955-w

**Published:** 2023-03-24

**Authors:** Jayesh Sheth, Siddharth Shah, Chaitanya Datar, Kaveri Bhatt, Pooja Raval, Aadhira Nair, Deepika Jain, Jhanvi Shah, Frenny Sheth, Harsh Sheth

**Affiliations:** 1 FRIGE’s Institute of Human Genetics, FRIGE House, Jodhpur Gam Road, Satellite, Ahmedabad India; 2 Royal Institute of Child Neurosciences, Vastrapur, Ahmedabad India; 3 Bharati Hospital and Research Centre, Dhankawadi, Pune, India; 4 KEM Hospital, Rasta Peth, Pune, India; 5 Shishu Child Development and Early Intervention Centre, Ahmedabad, India

**Keywords:** Multiple sulfatase deficiency, *FGE*, *SUMF1*, Ichthyosis, Case report

## Abstract

**Background:**

Multiple sulfatase deficiency (MSD) is a rare lysosomal storage disorder caused due to pathogenic variants in the *SUMF1* gene. The SUMF1 gene encodes for formylglycine generating enzyme (FGE) that is involved in the catalytic activation of the family of sulfatases. The affected patients present with a wide spectrum of clinical features including multi-organ involvement. To date, almost 140 cases of MSD have been reported worldwide, with only four cases reported from India. The present study describes two cases of late infantile form of MSD from India and the identification of a novel missense variant in the SUMF1 gene.

**Case presentation:**

In case 1, a male child presented to us at the age of 6 years. The remarkable presenting features included ichthyosis, presence of irritability, poor social response, thinning of corpus callosum on MRI and, speech regression. Clinical suspicion of MSD was confirmed by enzyme analysis of two sulfatase enzymes followed by gene sequencing. We identified a novel missense variant c.860A > T (p.Asn287Ile) in exon 7 of the *SUMF1* gene. In case 2, a two and a half years male child presented with ichthyosis, leukodystrophy and facial dysmorphism. We performed an enzyme assay for two sulfatases, which showed significantly reduced activities thereby confirming MSD diagnosis.

**Conclusion:**

Overall, present study has added to the existing data on MSD from India. Based on the computational analysis, the novel variant c.860A > T identified in this study is likely to be associated with a milder phenotype and prolonged survival.

**Supplementary Information:**

The online version contains supplementary material available at 10.1186/s12887-023-03955-w.

## Background

Multiple sulfatase deficiency (MSD) (OMIM#272200) is a rare lysosomal storage disorder with an incidence of 1 in 1.4 million newborns [1]. It occurs due to a defect in the *SUMF1* gene, located on chromosome 3p26.1, that encodes for formylglycine generating enzyme (FGE) [2–4]. FGE is involved in the posttranslational modification and catalytic activation of the family of sulfatase enzymes. The FGE protein catalyzes a cysteine residue shared by all sulfatase enzymes to form formylglycine. This step is critical for their enzymatic functions. As a result, any defect in the FGE can lead to deficiency in the sulfatase enzyme family along with increased levels of sulfated lipids and mucopolysaccharides inside the cells [5]. In humans, there are seventeen types of sulfatases of which nine are associated with MSD [6]. The FGE protein catalyzes a cysteine residue shared by all sulfatase enzymes to form formylglycine. This step is critical for their enzymatic functions. As a result, any defect in the FGE can lead to deficiency in the sulfatase enzyme family along with increased levels of sulfated lipids and mucopolysaccharides inside the cells [5].

MSD patients present with a variable clinical spectrum and are influenced by the severity of FGE protein instability and residual catalytic ability. The three types of MSD based on the clinical presentation have been described, that include, neonatal, late infantile, and juvenile. Of these, neonatal is the most severe form whereby affected individuals are presented with coarse facial features, skeletal abnormalities, neurologic deterioration, ichthyosis, and mental retardation [7]. Late infantile is the most common form of MSD and is characterized by normal development in early childhood and gradual regression of psychomotor skills during late childhood [7].

Overall, approximately 143 cases of MSD have been reported worldwide [8] with very few cases of MSD from India [9–11]. In the present study, we describe two cases of MSD from India with a novel *SUMF1* variant identified in one case along with the review of literature.

## Case presentation

**Fig. 1:**
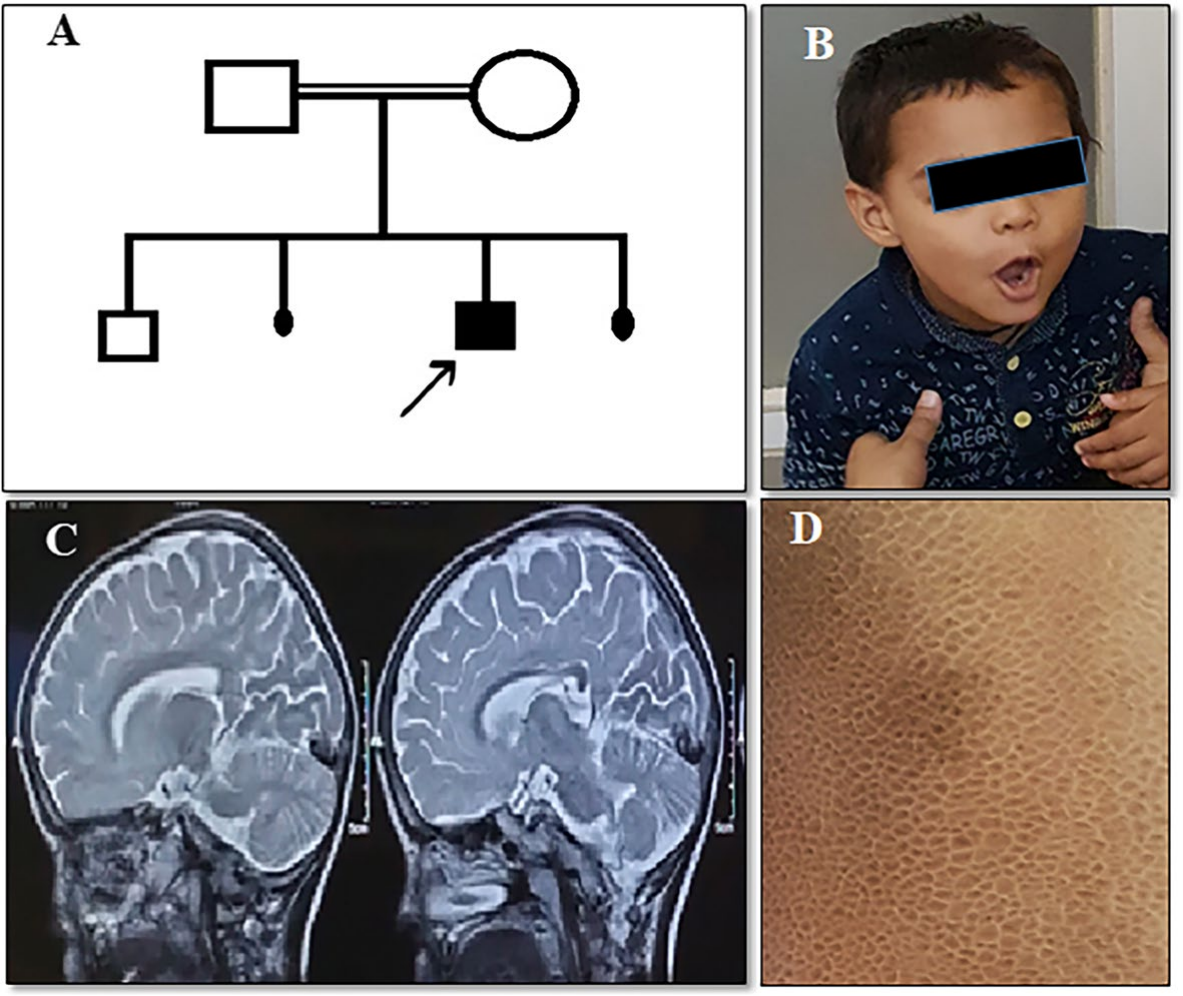
Case 1. **A**: Pedigree chart, proband is shown by arrow, **B**: Clinical photograph of the proband showing no facial dysmorphism, **C**: MRI of the proband showing mild thinning of posterior aspect of corpus callosum, **D**: Back region of the proband showing ichthyosis

Case 1: A male child born to a second-degree consanguineous Muslim couple from Ahmedabad, Gujarat presented at our clinic at 6 years of age (Fig. 1A).

**Fig. 2:**
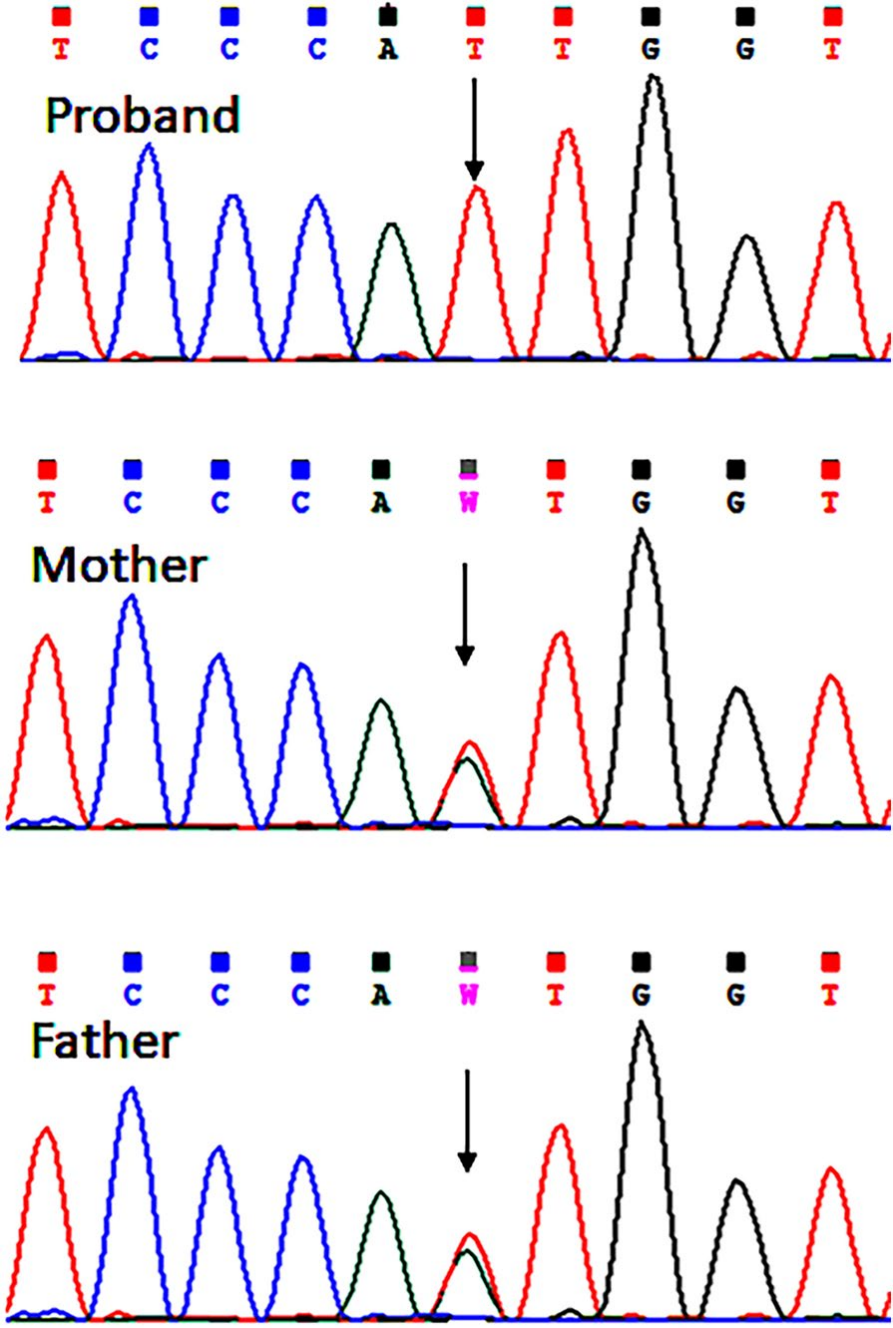
**A**, **B**, **C**: Sanger sequencing chromatogram of the proband and his parents

The index case was delivered at full term with a birth weight of 2250 g and had delayed cry. At the age of 9 months, he was hospitalized due to diarrhoea. The child contracted Tuberculosis (TB) at 4 years of age and received a TB regimen therapy for 9 months. He showed regression of speech at 2.5 years of age. He had a poor response to social interactions and had aggressive behaviour. At first, the child was referred to a paediatric neurologist who suspected a case of syndromic autism spectrum disorder. His sleep EEG study showed intractable epilepsy activity from the left occipital hemisphere with spread to the opposite hemisphere and diffuse damage from the right hemisphere. In addition, his MRI showed mild thinning of the posterior aspect of the body of the corpus callosum with no evidence of cerebral and cerebellar atrophy (Fig. 1B). Subsequently, the child was referred to our centre to carry out additional investigations. On examination, the remarkable observation noted was the presence of ichthyosis on both the legs, back, and abdomen (Fig. 1C). There was no facial dysmorphism observed. He could not speak and was restless and babbling. Primary investigations of karyotype and Fragile-X were normal in the proband. Considering abnormal MRI and the clinical indications, we suspected metachromatic leukodystrophy (MLD) or MSD. Hence, investigation for lysosomal enzymes arylsulfatase-A, arylsulfatase-B, and beta-galactosidase was carried out from leukocytes using 4-Mu fluorogenic synthetic substrates. Leukocytes were separated from the whole blood of the patient and all the enzyme assays were analyzed using spectrophotometric and fluorometric methods as described earlier [12, 13]. We found low level of arylsulfatase-A (0.08 nmol/hr/mg protein) (NR: 0.6- 4.99 nmol/hr/mg protein) and arylsulfatase-B (0.23 nmol/hr/mg protein) enzyme activities (NR: 0.61- 9.6 nmol/hr/mg protein) with normal activity of the beta-galactosidase enzyme. Considering low activity of both sulfatases: arylsulfatase-A and arylsulfatase-B, MSD was the likely diagnosis in the proband. Furthermore, for molecular confirmation, we performed *SUMF1* gene-sequencing study to determine the underlying causative variant. Genomic DNA was extracted from the peripheral blood of the proband using the desalting protocol [14]. Sanger sequencing of all the exons and exon–intron boundaries of the *SUMF1* gene (NM_182760.4) in the proband detected a homozygous missense variant c.860A > T (p.Asn287Ile) in exon 7. The variant c.860A > T has not been reported in the 1000 genomes and gnomAD databases. Also, in-silico predictions of the variant using Polyphen-2 and SIFT were found to be damaging. The variant was classified as likely pathogenic as per the ACMG-AMP guidelines and ClinGen framework [15–17] with the following criteria – PM1 (supporting), PM2 (moderate), PP3 (supporting) and, PS3 (moderate). Following this, a parental study was carried out that confirmed them to be heterozygous carriers for the same variant in the *SUMF1* gene (Fig. 2).

Case 2: A two and half-year-old male child, born to a non-consanguineous Muslim couple was referred to our clinic. The notable features included ichthyosis and facial dysmorphism. His MRI study showed leukodystrophy, which is a classical observation seen in patients with MLD and Krabbe disease. Based on these observations, child was investigated for lysosomal enzyme activity from leukocytes for arylsulfatase-A. However, due to presence of ichthyosis, MSD was also suspected and hence activity of another sulfatase: N-acetyl-galactosamine-6-sulfate-sulfatase was assessed as per the method earlier described [18]. The proband showed significantly low activity of arylsulfatase-A enzyme (0.045 nmol/hr/mg protein) and N-acetyl-galactosamine-6-sulfate-sulfatase enzyme (0.041 nmol/hr/mg protein) (NR: 2.8- 21.6 nmol/hr/mg protein) with the normal activity of beta-galactosidase enzyme. This confirmed the diagnosis of MSD in the proband. However, at the time of the investigation, the patient’s family refused to carry out further molecular study and hence the causative variant was not identified. Furthermore, the family was lost to follow-up and hence there is a lack of clinical, MRI pictures and details regarding the progression of the disease condition in order to comment on the severity.

## Discussion and conclusion

MSD is an ultra-rare lysosomal storage disorder characterized by a deficiency of the FGE protein. The key presenting features of MSD are neurological complications, developmental delay, skeletal and dermatological abnormalities [8]. Of these, ichthyosis is the most frequently reported sign (~ 71% cases) followed by organomegaly (57% cases) and dysostosis multiplex (56% cases). Ichthyosis was present in both our cases, which is consistent with the earlier observation [8]. Facial dysmorphism, which is another common sign in MSD patients, was seen in only one of the two cases. Strikingly, skeletal abnormalities and organomegaly, which are generally common in MSD patients, were absent in both cases. Hijazi et al. also made a similar observation where none of the patients showed organomegaly and only 2/6 patients had a skeletal abnormality. This explains the wide clinical variability observed in MSD cases. The common MRI findings in MSD patients include leukodystrophy, cerebral and cerebellar atrophy, and hydrocephalus [19]. Interestingly, both our patients showed abnormal MRI with thinning of the corpus callosum in one case and leukodystrophy in another. For the first time, Hijazi et al. recorded autistic features in MSD patients [19]. We noted a similar finding in the proband from case 1. This suggests that autism spectrum disorder is likely to be a secondary manifestation arising due to MSD and often misdiagnosed as autism.

MSD is classified into three types depending on the age of onset and severity. Schlotawa and his group analysed the correlation between the onset of key signs and the survival age of the patient [8]. They showed neurological and skeletal signs before 24 months of age to be negatively associated with survival. As opposed to this, the presence of only ichthyosis after 36 months correlated with longer survival. Both of our patients are likely to have longer survival as both are alive to date. Yet, it is critical to note that cardiovascular and respiratory involvement is associated with reduced survival and is independent of onset age. Also, respiratory complications have been the most common cause of death in the case of MSD patients [8]. Though, none of our patients have shown respiratory complications yet. A recent study by Beck-Wödl et al. compared the clinical course of two patients with MSD to a broader cohort of MLD patients. Their observations suggested that patients with MSD show early onset of motor symptoms as compared to patients with juvenile MLD, but the disease progression is slow as compared to the juvenile MLD patients [20]. This is similar to what we observed for proband in case 1. Thus, this can be a key clinical observation in differentiating MSD patients from MLD patients.

Detecting elevated levels of glycosaminoglycan’s and other sulphated sugars in the patient’s urine sample can aid in the diagnosis of MSD and also to rule out arylsulfatase-A pseudo deficiency which is a common observation in ~ 7–12% of healthy population [21]. However, in the present study, these investigations were not carried out, as both cases had a strong clinical suspicion and positive lysosomal enzyme study. Based on this, MSD was considered as the most likely differential diagnosis.

The primary diagnosis of MSD can be carried out by lysosomal enzyme study followed by confirmative genetic investigation. It is important to note that the method of diagnosis does not affect patient survival in MSD; hence, either of the options can be used for the purpose of diagnosis [8]. The key advantage in cases where a molecular diagnosis has been made is that prenatal testing can be offered for future pregnancies. Biochemical diagnosis is achieved by reduced activity of two or more sulfatases whereas molecular testing determines the causative variant in the *SUMF1* gene. At the molecular level, the clinical heterogeneity is explained by the amount of residual enzyme activity and the degree of protein stability [22]. In proband of case 1, we have identified a novel missense variant c.860A > T in the *SUMF1* gene. So far, a majority of missense mutations have been reported, followed by nonsense and splicing mutations majority in the C-terminal domain of the FGE protein [22]. As the variant identified in our case is novel, we have carried out a computational analysis to determine its pathogenicity. We performed a structural study of the mutant SUMF1 using the crystallographic structure of FGE (PDB ID: 1Y1E) as the template. The native and mutant structure was modelled using a web server Swiss Model of Expasy. The two models were superimposed and aligned using the tool UCSF Chimera to understand the effect of the variant [23]. The superimposed model suggests that there is no significant conformational change in the loop region due to the presence of mutant amino acids Ile at position 287 of the protein (Supplementary Fig. 1). However, due to the change of a hydrophilic amino acid asparagine to a hydrophobic amino acid isoleucine, there is likely to be a disruption in protein binding resulting in overall instability of the protein.

High percentage of MSD cases have been reported from the USA, Saudi Arabia and, Turkey with thirty-one, twenty-six and, fifteen cases respectively. While from India only four cases have been reported with molecular study in one of them showing compound heterozygous missense mutation in exon 3 and insertion mutation in exon 5 as shown in Table 1. The majority of mutations have been identified in exon 5, 6 and, 9 suggesting them as the hot spot region. However, the present study has identified a missense mutation in exon 7 suggesting the possibility of ethnographic variability. Previously, a couple of studies have analyzed the functional effects of a subset of SUMF1 variants on the FGE protein [24–26] with the correlation between genotype and phenotype in MSD depending on protein stability and residual activity of mutant FGE protein. However, as more novel variants are being identified in the *SUMF1* gene, functional and computational studies to predict the consequence of these variants at the molecular and clinical level need to be performed. The milder phenotype in case one in the present study could be attributed to the missense mutation affecting only protein stability. Thus, present study will add to the existing understanding of genotype–phenotype correlation in MSD and can aid in predicting disease severity and providing genetic counselling to the affected families.

**Table 1:** Review of mutation study of multiple sulfatase deficiency

Sr. no	Exon	cDNA position	Amino acid position	Coding impact	Reference
1	1	c.1A > G	p. Met1Val	Missense	[7]
2	1	c.2 T > G	p. Met1Arg	Missense	[7]
3	1	c.58C > T	p.Leu20Phe	Missense	[7]
4	1	c.132_133insG	-	Frameshift	[7]
5	1	c.156delC	p.Cys52TrpfsTer57	Frameshift	[7]
6	1	c.191C > A	p.Ser64Ter	Nonsense	[3, 7]
7	1	c.243delC	p.Ile94SerfsTer15	Frameshift	[7]
8	2	c.276delC	p.Gly82GlufsTer27	Frameshift	[7]
9	2	c.305G > T	p.Gly102Val	Missense	[2]
10	2	c.389A > G	p.Glu130Gly	Missense	[8]
11	2	c.390A > G	p.Glu130Asn	Missense	[27]
12	2	c.392 T > G	p.Val131Gly	Missense	[8]
13	3	c.451A > G	p.Lys151Glu	Missense	[7, 9]
14	3	c.463 T > C	p.Ser155Pro	Missense	[1, 2, 7]
15	3	c.464G > A	p.Ser155Phe	Missense	[2]
16	3	c.519 + 4A > G	-	splice site	[7]
17	3	c.519 + 5_c.519 + 8del	-	Frameshift	[7]
18	Intron 3	IVS3 + 5-8del	-	Splice site	[7]
19	4	c.520_954dup	p.V174-P318dup	-	[7, 19]
20	4	c.529G > C	p.Ala177Pro	Missense	[2, 20, 25, 26]
21	4	c.536G > C	p.Trp179Ser	Missense	[2, 7]
22	4	c.539G > T	p.Trp180Leu	Missense	[2]
23	5	c.602 + 1G > A	-	Splice site	[7]
24	5	c.603-2delA	-	Frameshift	[7]
25	5	c.640G > A	p.Ala214Thr	Missense	[2]
26	5	c.653G > A	p.Cys218Tyr	Missense	[7]
27	5	c.661delG	p.Ala221GlnfsTer47	Frameshift	[7]
28	5	c.670C > T	p.Arg224Trp	Missense	[7]
29	5	c.671G > A	p.Arg224Gln	Missense	[7]
30	5	c.690-691insT	-	Frameshift	[7, 9]
31	5	c.700A > T	p.Ser234Arg	Missense	[7]
32	5	-	p.Ser236Ter	Nonsense	[7, 19]
33	6	c.725 + 1G > C	-	Splice site	[7]
34	6	c.731 T > C	p.Phe244Ser	Missense	[4]
35	6	c.739G > C	p.Gly247Arg	Missense	[1, 2, 4, 8, 19]
36	6	c.748delC	p.Leu250CysfsTER18	Frameshift	[7]
37	6	c.776A > G	p.Asn259Ser	Missense	[2, 7]
38	6	c.776A > T	p.Asn259Ile	Missense	[7, 19]
39	6	c.777C > A	p.Asn259Lys	Missense	[7]
40	6	c.785A > G	p.Gln262Arg	Missense	[7]
41	6	c.788G > T	p.Gly263Val	Missense	[1, 7, 19]
42	6	-	p.Ser265Ter	Nonsense	[7]
43	6	c.797C > T	p.Pro266Leu	Missense	[2, 21]
44	6	c.817G > A	p.Asp273Asn	Missense	[2]
45	6	c.818A > G	p.Asp273Gly	Missense	[3, 7]
46	6	c.818A > T	p.ASP273Val	Missense	[7]
47	6	c.836C > T	p.Ala279Val	Missense	[2, 19]
**48**	**7**	**c.860A > T**	**p.Asn287Ile**	**Missense**	**Present study**
49	7	c.890A > C	p.Asn297Thr	Missense	[2]
50	7	c.893 C > A	p.Ala298Glu	Missense	[7, 19]
51	Intron 7	IVS7 + 5G > T	-	Splice site	[7]
52	8	c.979C > T	p.Arg327Ter	Nonsense	[7]
53	8	c.1006 T > C	p.Cys336Arg	Missense	[7]
54	8 & 9	28 kb deletion	-	Frameshift	[4]
55	9	c.1018 T > C	p.Tyr340His	Missense	[19]
56	9	c.1029G > C	p.Arg343Ser	Missense	[19]
57	9	c.1033C > T	p.Arg345Cys	Missense	[1, 7]
58	9	c.1034G > T	p.Arg345His	Missense	[2]
59	9	c.1038 T > G	p.Cys346Trp	Missense	[7]
60	9	-	p.Arg348Pro	Missense	[7]
61	9	c.1043C > T	p.Ala348Val	Missense	[2, 20]
62	9	c.1045C > T	p.Arg349Trp	Missense	[1, 8]
63	9	c.1045C > G	p.Arg349Gly	Missense	[7]
64	9	c.1046G > A	p.Arg349Gln	Missense	[1]
65	9	c.1076C > A	p.Ser359Ter	Nonsense	[7]
66	9	c.1091G > A	p.Arg364His	Missense	[2]
67	9	c.1090C > T	p.Arg364Cys	Missense	[7]

There is a high degree of clinical variability seen in MSD patients, with ichthyosis and developmental delay being the common signs. At present, there is no treatment available, however, the involvement of a multidisciplinary team can aid in the holistic management of MSD patients. It is evident from the data in the literature that exon 6 of the *SUMF1* gene is the hotspot for mutation; hence, this exon can be preliminary screened in MSD patients to identify the causative variant. Lastly, the variant identified in the present study, c.860A > T, based on the computational analysis is likely to have a relatively milder phenotype and prolonged survival.

## Supplementary Information

**Additional file 1: Figure 1. **Superimposed native structure (blue) and mutant structure (brown) of the FGE protein produced using UCSF chimera. The purple highlighted portion is the wild-type residue and the blue highlighted portion is the mutant residue.

## Data Availability

Not applicable.

## Abbreviations

MSD: Multiple sulfatase deficiency
SUMF1: Sulfatase Modifying Factor1
FGE: Formyl glycine generating enzyme
TB: Tuberculosis
MRI: Magnetic resonance imaging
EEG: Electroencephalography
DNA: Deoxyribonucleic acid
PDB: Protein data bank
