# Supplementary figures and images for: Late infantile form of multiple sulfatase deficiency with a novel missense variant in the SUMF1 gene: case report and review
Source: BMC Pediatr. 2023 Mar 24;23:133. doi: 10.1186/s12887-023-03955-w (PMC10037891; doi:10.1186/s12887-023-03955-w)

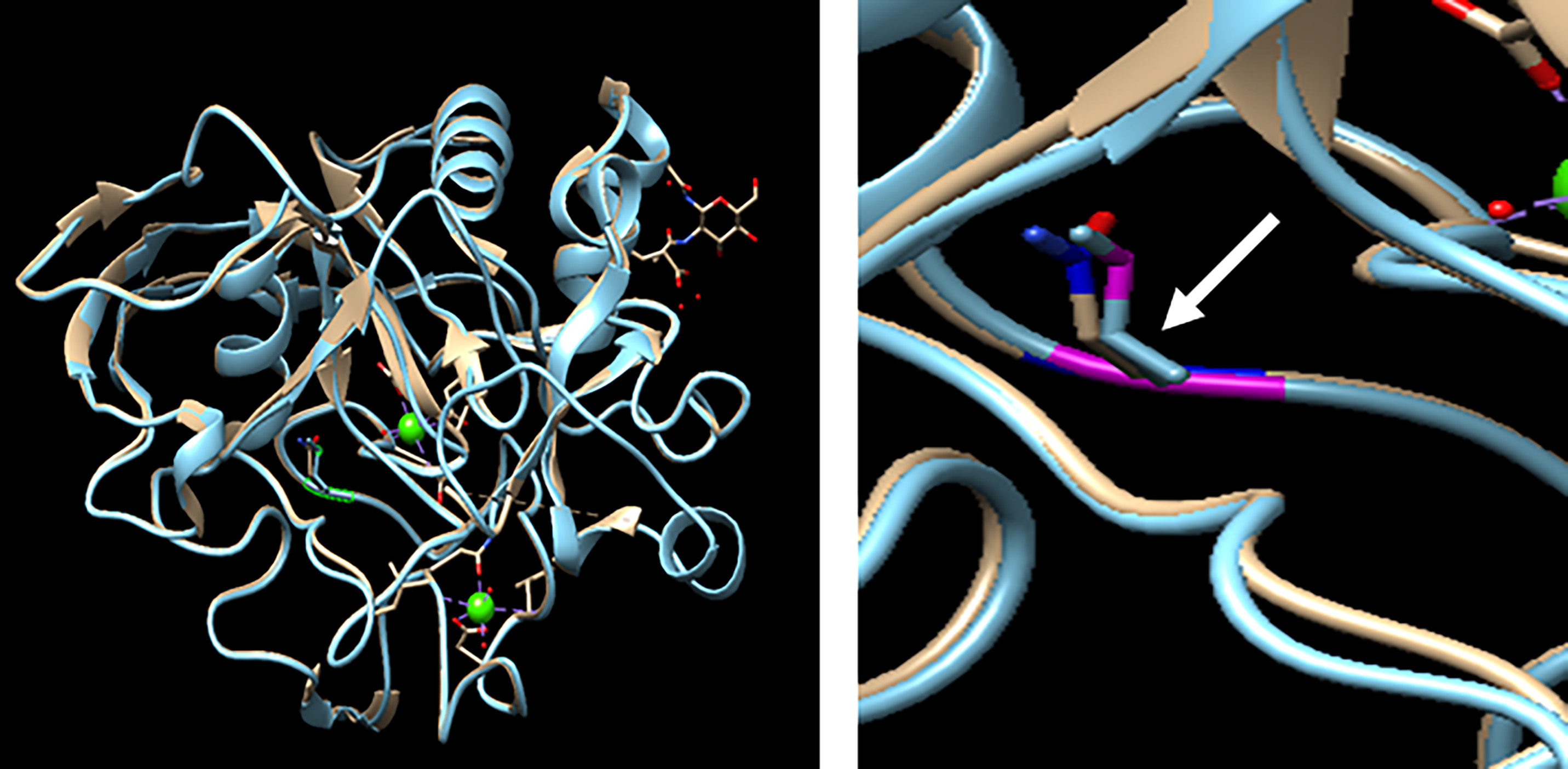

Supplement: Supplementary file 1 — Additional file 1: Figure 1. Superimposed native structure (blue) and mutant structure (brown) of the FGE protein produced using UCSF chimera. The purple highlighted portion is the wild-type residue and the blue highlighted portion is the mutant residue. [file 12887_2023_3955_MOESM1_ESM.jpg]
